# Pedestrians' perceptions of route environments in relation to deterring or facilitating walking

**DOI:** 10.3389/fpubh.2022.1012222

**Published:** 2023-06-06

**Authors:** Dan Andersson, Lina Wahlgren, Peter Schantz

**Affiliations:** ^1^The Research Unit for Movement, Health and Environment, Department of Physical Activity and Health, The Swedish School of Sport and Health Sciences, GIH, Stockholm, Sweden; ^2^Section of Sustainable Health, Department of Public Health and Clinical Medicine, Umeå University, Umeå, Sweden

**Keywords:** walking, route environment, conflicts, aesthetics, greenery, noise, vehicle speed, environmental unwellbeing – well-being

## Abstract

**Background:**

Every walk takes place in a route environment, and it can play an important role in deterring or facilitating walking, and will always affect the environmental unwell-well-being of pedestrians. The aim of this study is to illuminate which the important route environmental variables are in this respect. The focus is, therefore, on pedestrians' perceptions of route environmental variables and how they relate to overall appraisals of route environments as hindering–stimulating for walking and unsafe–safe for reasons of traffic.

**Methods:**

Commuting pedestrians in the inner urban area of Stockholm, Sweden (*n* = 294, 49.5 ± 10.4 years, 77% women), were recruited *via* advertisements. They evaluated their commuting route environments using a self-report tool, the Active Commuting Route Environment Scale (ACRES). Correlation, multiple regression, and mediation analyses were used to study the relationships between the variables and the outcome variables.

**Results:**

Aesthetics and greenery appear to strongly stimulate walking, whereas noise, a proxy for motorized traffic, hinders it. Furthermore, aesthetics is positively related to traffic safety, whereas conflicts have the opposite role. Conflicts is an intermediate outcome, representing several basic environmental variables, some of which were directly and negatively related to unsafe–safe traffic.

**Conclusion:**

Route environmental variables appear to be potent factors in deterring or facilitating walking. This knowledge is of importance for policymakers and urban planners when designing route environments with the aim of attracting new pedestrians, and simultaneously stimulating those who already walk to keep on.

## 1. Introduction

Every walk takes place in a route environment that induces a sense of unwellbeing–well-being for the person who walks in it (1). In line with the broad definition of health by WHO (2), it thereby influences aspects of our health.

It is reasonable that the degree of environmental unwellbeing–well-being that a route generates will influence our willingness to walk and our associated decisions. In case we choose to walk, the route environments can, hypothetically, affect our walking behaviors in terms of route choice, duration, frequency, and intensity, as well as to the degree that behaviors are sustained over time or not. Nonetheless, to study how environmental variables influence the spectrum of environmental unwellbeing–well-being while walking is important, independently of whether it affects our walking behavior.

**Figure 1:**
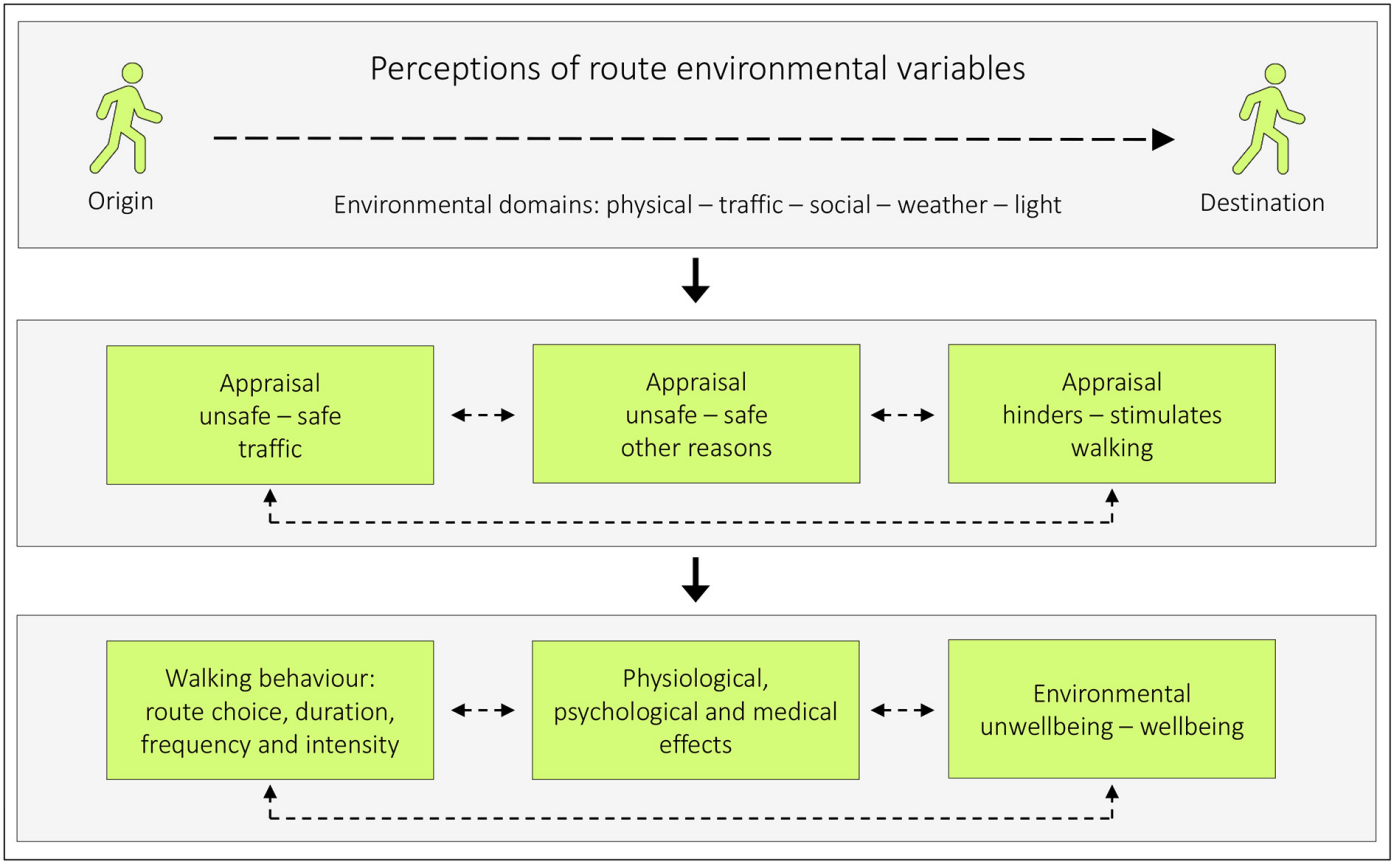
A model illustrating how perceptions of route environmental variables may affect pedestrians [modified from (1), p. 150, (7), p. 6]. Route environments consist of different environmental domains: the physical environment (stationary objects such as buildings and trees), the traffic environment (mobile objects such as cars and pedestrians), the social environment (interaction between individuals), weather (wind, rain etc.), and light conditions (natural and artificial). Perceptions of these domains can influence how we appraise unsafety – safety due to traffic, unsafety – safety due to other reasons (such as harassment), and how we appraise the environment with respect to if it is hindering – stimulating for walking. These appraisals can, consecutively, affect our walking behavior (such as route choice and the amount of walking), have physiological, psychological, and medical effects, and will always affect our environmental unwellbeing – wellbeing. The bidirectional hatched lines between the boxes indicate potential mutual relationships.

If environmental unwellbeing–well-being is viewed as a final outcome (refer to the conceptual model, Figure 1), at least three intermediate outcomes are constituents of it, namely unsafety–safety for reasons of traffic, ambient conditions that hinder–stimulate walking, and other reasons. Route environmental variables within five different domains can influence these intermediate outcomes and the final outcomes.

This conceptual framework is based on experience from long-term educational practice by one of the authors (PS). Further support comes from the scientific literature. Safety (for different reasons) is an important issue among those who walk (3–6). In addition, a need for the supplementary outcome *hinders–stimulates walking* has been identified [(7), p. 25]. Furthermore, Panter et al. (8) assessed associations between changes in perceptions of the environment en route to work and changes in the level of active commuting. Commuters who reported that it became less pleasant to walk, or perceived more danger while crossing a road, recorded a net decrease in walking. Another example is that traffic volume and speed combined have been described as a barrier to walking affecting hedonic well-being negatively (9). This would in such case refer to the psychological effect in the model. Physical activity can also affect well-being (10), and it is, therefore, important to separate that effect from how walking routes affect environmental unwellbeing–well-being.

Many environmental assessment tools related to walking have been developed. Examples of these include the Neighborhood Environment Walkability Scale (NEWS) by Saelens et al. (11), the Twin Cities Walking Survey (TCWS) by Oakes et al. (12), and the Saint Louis Environment and Physical Activity Instrument by Brownson et al. (13). They all have questions about aspects of the built and traffic environments, using primarily Likert scales of 4-point response.

However, none of them have had the purpose of studying the relations between route environmental variables and the intermediate outcomes unsafety–safety due to traffic or hindering–stimulating for walking (cf. Figure 1). It was therefore considered of great value to develop and evaluate a specialized tool for those purposes: The Active Commuting Route Environment Scale (ACRES) (14, 15). The ACRES is a self-report tool that assesses cycling and pedestrian commuters' perceptions and appraisals of their individual commuting route environments using 15-point response scales.

The aim of this study is to illuminate the role of route environmental variables in relation to the outcomes that *hinders–stimulates walking* and *unsafe–safe traffic*, thereby mirroring factors deterring or facilitating walking. Another focus is to explore if there are (i) intermediate outcome variables among the predictors, which represent more basic variables, and (ii) direct or mediating effects between certain variables. We have, therefore, studied male and female commuters (*n* = 294) rating their own, self-chosen, commuting routes in the inner urban parts of Stockholm, Sweden.

## 2. Methods

### 2.1. Procedure and participants

This study is part of a research project entitled Physically Active Commuting in Greater Stockholm (PACS). Active commuters were recruited *via* two large newspapers in Stockholm (Dagens Nyheter and Svenska Dagbladet) toward the end of May and early June 2004. Inclusion criteria were: (a) being at least 20 years old; (b) living in Stockholm County (excluding the municipality of Norrtälje), and (c) walking and/or cycling the entire distance to one's place of work or study at least once a year. It was emphasized that individuals with all commuting distances were welcome to participate.

The advertisement led to 2,148 volunteers. The first questionnaire, the Physically Active Commuting in Greater Stockholm Questionnaire (PACS Q1), was distributed in September 2004. The response frequency was 94% (*n* = 2,010). A second questionnaire, the PACS Q2, was distributed in May 2005. The response frequency was 92% (*n* = 1,819). The participants were cyclists, pedestrians, or dual-mode commuters, that is, those who sometimes cycle and sometimes walk. They commuted in the inner urban or suburban–rural areas of Greater Stockholm, or in both of these areas. For further information about the recruitment process, refer to [(15–17), pp. 65–66].

We have exclusively used data on walking commuting from the inner urban area in this study. Some of the participants (26.2%) commuted in the suburban area as well but only their inner urban data have been used. A previous study has shown that cycle commuting in both areas does not affect ratings in either of the areas (15). After cleansing and editing the data, 294 participants (77% women) were included in the analyses: 56.5% were pedestrians and 43.5% were dual-mode commuters. For further descriptive characteristics of the participants, refer to Table 1. The Ethics Committee North of the Karolinska Institute at the Karolinska Hospital approved the study (Dnr 03-637). The participants gave their informed consent.

**Table 1:** Descriptive characteristics of participants (*n* = 282–294)*.

Females**, %	77
Age in years**, mean ± SD	49.5 ± 10.4
Weight in kg, mean ± SD	68.4 ± 10.6
Height in cm, mean ± SD	171.1 ± 8.2
Body mass index, mean ± SD	23.3 ± 2.7
Gainful employment, %	97
Educated at university level**, %	79
Income**:	
≤ 25 000 SEK*** a month, %	37
25 001–30 000 SEK*** a month, %	27
≥ 30 001 SEK*** a month, %	35
Participant and both parents born in Sweden, %	80
Having a driver's license, %	89
Usually access to a car, %	56
Leaving home 7–9 a.m. to walk to work or study place, %	80
Leaving place of work or study 4–6 p.m. to walk home, %	73
Number of walking-commuting trips per year****, mean ± SD	278 ± 162
Overall physical health either good or very good, %	77
Overall mental health either good or very good, %	85

*294 individuals have complete data regarding ACRES and the four background variables that are used in the analyses; **Descriptive characteristics used as a predictor variable in the multiple regression analyses; ***SEK, Swedish crowns/kronor, year 2005: €1 ≈ 9 SEK; US$1 ≈ 8 SEK; ****The number of walking-commuting trips per year is based on 224 participants, whereof four individuals had abnormally high values, which therefore were substituted with mean values for the remainder of the participants. The low response rate is due to missing values in one or more of the 12 months leading to exclusion in the sum score.

### 2.2. Descriptive characteristics of the participants

Data on sex, age, weight, height, employment, and the number of pedestrian-commuting trips per month were obtained from the PACS Q1. Active commuting trips per year were calculated by adding each of the 12 months' average trip frequency per week, dividing by 12, and multiplying by 52. Educational levels, income, ethnicity, having a driver's license, having access to a car, time leaving home to walk to work, and overall physical and mental health were obtained from the PACS Q2, refer to Table 1.

### 2.3. The physically active commuting in Greater Stockholm questionnaire (PACS Q1 and Q2)

The PACS Q1 and PACS Q2 are self-report questionnaires in Swedish, which include questions about background factors and different aspects of active commuting. They consist of 35 and 68 items, respectively. The ACRES is included in the PACS Q2. PACS Q1 and Q2, translated into English, can be found as supporting information here (18).

#### 2.3.1. The active commuting route environment scale (ACRES)

To explore relationships between individuals actively transporting themselves between home and work and the route environment, the ACRES was developed and evaluated (14, 15). The pedestrian version of the scale consists of 13 items, refer to Table 2. Two of these items, *hinders–stimulates walking* and *unsafe–safe traffic*, are outcome variables but can, in relation to each other and the other items, also act as predictor variables.

**Table 2:** The Active Commuting Route Environment Scale (ACRES) for pedestrians.

**Question**	**15-point response scale**		**Variable name**
	**1**	**15**	
1. Do you think that, on the whole, the environment you walk in stimulates/hinders your commuting?	Hinders a lot	Stimulates a lot	Hinders–stimulates walking*
2. How do you find the exhaust fume levels along your route?	Very low	Very high	Exhaust fumes
3. How do you find the noise levels along your route?	Very low	Very high	Noise
4. How do you find the flow of motor vehicles (number of cars) along your route?	Very low	Very high	Flow of motor vehicles
5. How do you find the speeds of motor vehicles (taxis, lorries, ordinary cars, buses) along your route?	Very low	Very high	Speeds of motor vehicles
6. How do you find the congestion levels caused by the number of pedestrians along your route?	Very low	Very high	Congestion: pedestrians
7. How do you find the occurrence of conflicts between you as a pedestrian and other road users (including pedestrians) along your route?	Very low	Very high	Conflicts
8. How unsafe/safe do you feel in traffic as a pedestrian along your route?	Very unsafe	Very safe	Unsafe–safe traffic*
9. How do you find the availability of greenery (natural areas, parks, planted items, trees) along your route?	Very low	Very high	Greenery
10. How ugly/beautiful do you find the surroundings along your route?	Very ugly	Very beautiful	Ugly–beautiful
11. To what extent do you feel that your walking trip is made more difficult by the course of the route? For example, a course with many sharp turns, detours, changes in direction, side changeovers, etc.	Very little	Very much	Course of the route
12. To what extent do you feel that your walking trip is made more difficult by hilliness? Base this on the route to and from your place of work/study.	Very little	Very much	Hilliness
13. To what extent do you feel that your progress in traffic is worsened by the number of red lights during your trip to your place of work/study?	Very little	Very much	Red lights

This is a translation of the original ACRES in Swedish. *Outcome variable.

**Figure 2:**
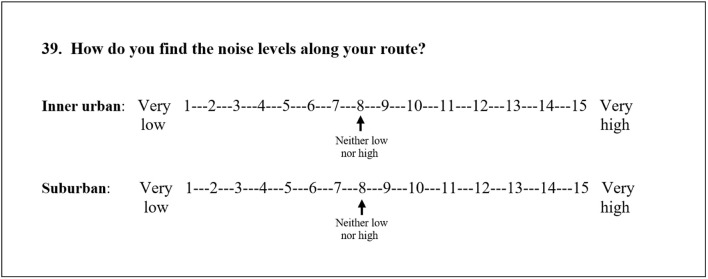
The respondent is asked to encircle the figure that matches the experience, and distinguish the experience in the inner urban area, from the experience in the suburban area. The 15-point response scales, with adjective opposites, have numbered continuous lines, i.e., whole numbers from 1 to 15. In addition, number 8, a neutral option, is labeled, for example, *neither low nor high*. This is a translation of the original question in Swedish.

Each item in the ACRES considers the inner urban area of Stockholm, Sweden, and the suburban areas surrounding it within Stockholm County, separately. For an example of an item, refer to Figure 2. The pedestrians were instructed to recall and rate their overall experience of their self-chosen route environments based on their active commuting to their place of work or study during the previous 2 weeks. A more detailed description of the development of the scale, as well as of its validity and reliability for cyclists, has been reported elsewhere (14, 15). In brief, the ACRES was characterized by considerable criterion-related validity and reasonable test–retest reproducibility.

### 2.4. Study area

**Figure 3:**
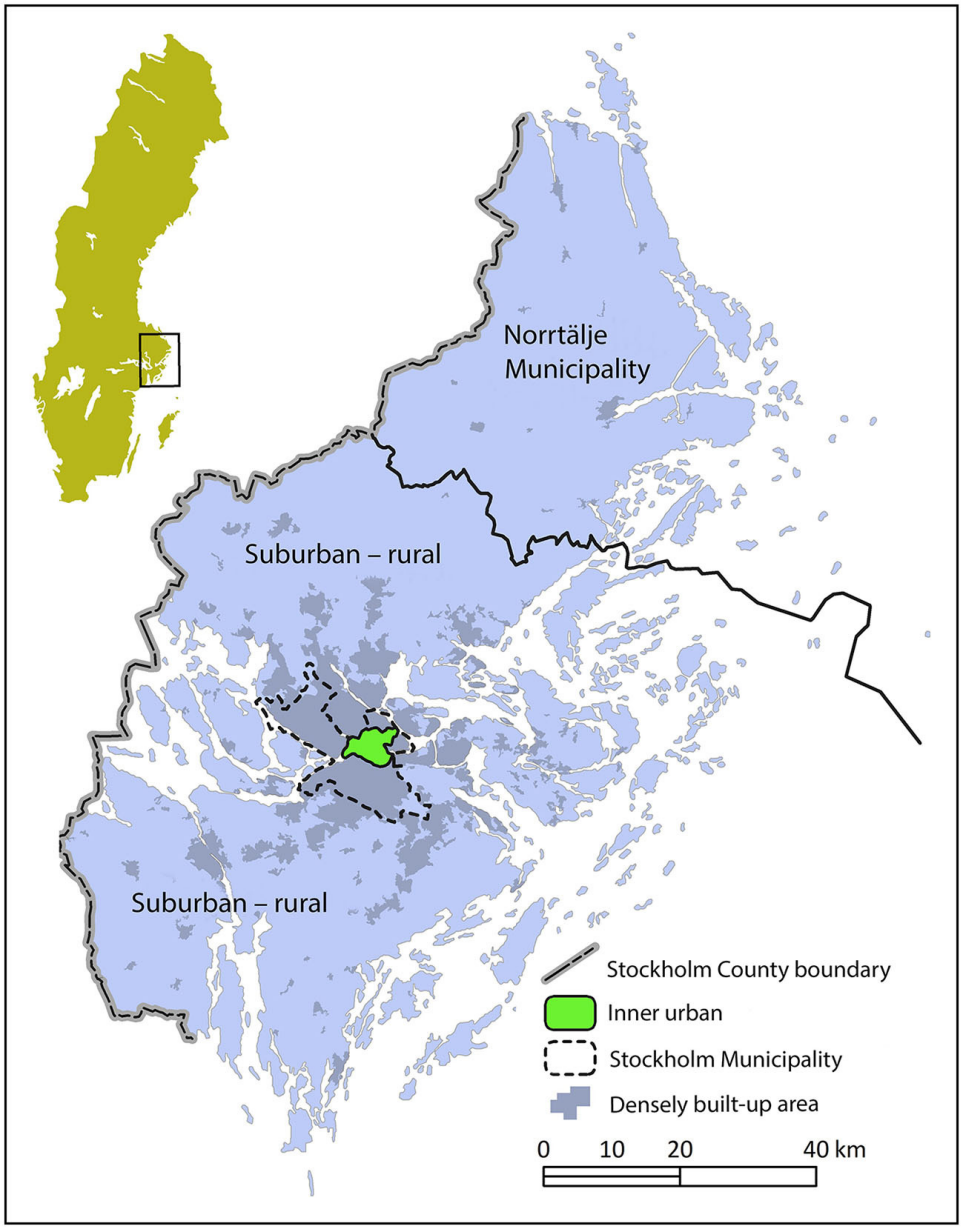
Map over Sweden and the Stockholm County, with the inner urban study area of the Municipality of Stockholm in green. The markings for the densely built-up areas illuminate the conditions in 2010. North is at the top of the image.

**Figure 4:**
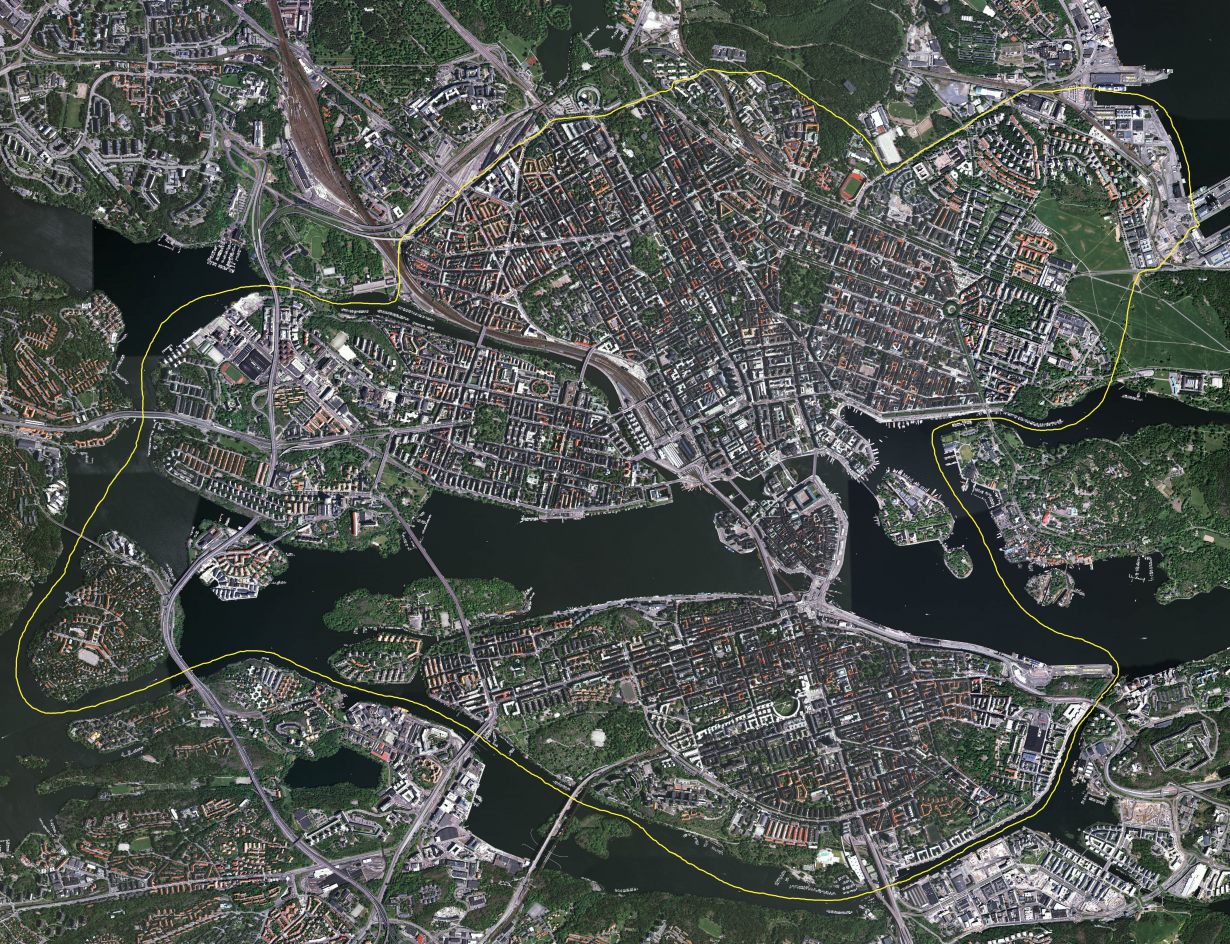
Aerial view of the inner urban study area of Stockholm in 2005. North is at the top of the image (Copyright: Lantmäteriverket, Gävle, Sweden, 2011; Permission 81055230).

The commuting route environments are located in the inner urban area of Stockholm, the capital of Sweden, in the center of a metropolitan area with, at the time, 1.9 million inhabitants (see Figure 3). The inner urban area, in our geographical division of the city, includes the city sections of Gamla stan, Södermalm, Kungsholmen, Vasastan, Norrmalm, and Östermalm. The inner urban area, with blocks organized in grid-like streetscapes (Figure 4), constitutes a different environment compared to the suburban and rural parts of Stockholm County. A more detailed description of the study area can be found in Wahlgren [(19), pp. 62–63].

### 2.5. Statistical analyses

Questionnaire data were entered in the Statistical Package for the Social Sciences and analyzed in version 27.0 (IBM SPSS Inc., Somers, NY, USA). All data from PACS Q2, which includes ACRES, were checked for accuracy. Some participants were excluded, mainly because of incorrect or incomplete ACRES data. Participants with no missing ACRES values and no missing values regarding the four descriptive characteristics were used for the analyses. The used descriptive characteristics were *sex* (dichotomous categorical variable: females = 0 and males = 1), *age* (continuous variable), *education* (categorical variable coded as dichotomous: educated at university level = 0 and not educated at university level = 1), and *income* (categorical variable coded as three categories: ≤ 25,000 SEK/month = 1, 25,001–30,000 SEK/month = 2 and ≥30,001 SEK/month = 3; SEK = Swedish crowns/kronor, year 2005: €1 ≈ 9 SEK; US$1 ≈ 8 SEK) (see Table 1).

Differences between ratings of ACRES between male and female participants were examined with independent *t*-tests. Correlations between the ratings of the 13 items in the ACRES were assessed using Pearson's correlation coefficient (r). Multiple regression analysis (MRA) was chosen to explore the relationships between the predictor variables and the outcome variables. The background variables *sex, age, education*, and *income* were included in the MRA.

Six MRA were run (Models 1–6); Models 1 and 2 had *hinders–stimulates walking* as an outcome and the items in the ACRES as predictors (Model 1 excluded *unsafe–safe traffic* and Model 2 included *unsafe–safe traffic*). The reason for including *unsafe–safe traffic* as a predictor, a variable that normally acts as an outcome variable, is its possible association with the outcome variable *hinders–stimulates walking*. Model 1 can be found in the Results section and Model 2 can be found in Supplementary Table A1. Models 3 and 4 had *unsafe–safe traffic* as an outcome and the items in the ACRES as predictors (Model 3 excluded *hinders–stimulates walking* and Model 4 included *hinders–stimulates walking*). The reason for including *hinders–stimulates walking* as a predictor, a variable that normally acts as an outcome variable is its possible association with the outcome variable *unsafe–safe traffic*. Model 3 can be found in the Results section and Model 4 can be found in Supplementary Table A2. Model 5 explores *conflicts* and their relations to relevant items in the ACRES. Model 6 explores the relation between *aesthetics* (variable name: *ugly–beautiful*) and all other items in the scale except the two outcome variables.

Five mediation analyses (MA) using PROCESS, a macro designed for SPSS, were run to have a closer look at the impact of one of the intermediate outcomes. PROCESS, written by Professor Andrew F. Hayes, can be downloaded to SPSS from www.processmacro.org (20). To test the indirect effect, we used 5,000 bootstrap samples to generate a confidence interval.

The linearity between the environmental variables (the items in the ACRES) was assessed visually by means of boxplots, error bars and scatterplots. All environmental variables showed reasonable linearity and were therefore used in the analyses. Furthermore, the distribution of the variables was approximately normal according to visual analyses of the boxplots, error bars and scatterplots.

Correlation analyses between the environmental variables were assessed with Pearson's correlation coefficient. The correlations were, in absolute values, r ≤ 0.774 (correlations between the background variable age and the predictor variables were, in absolute values, r ≤ -0.137). According to Tabachnik and Fidell, multicollinearity can be a problem with correlations > 0.90 [(21), pp. 88–90], and according to Field, an approximate method for identifying multicollinearity, that will miss subtler forms, is to pay attention to predictors that correlate very highly, values of *r* above 0.80 or 0.90 [(22), p. 402].

Due to a previous study (23), where *noise* protruded as the dominant negative predictor variable among the four variables related to motorized traffic in the ACRES, we have chosen to include *noise* as a proxy in relation to *hinders–stimulates walking*. With respect to our other outcomes, *unsafe–safe traffic, noise*, and *speeds of motor vehicles* will, due to the same rationale, act as proxies for that quartet of variables.

Our explorative approach included, as stated above, a search for intermediate outcomes among the predictors in this study. This led to an MRA with *conflicts* as an outcome and relevant items in the ACRES as predictors (Model 5). For this purpose, we analyzed all four traffic variables as predictors (not shown). It resulted in choosing *noise* and *speeds of motor vehicles* as proxies for the quartet of traffic variables in relation to *conflicts* as the outcome. Thereafter, we also included the following predictors: *noise, speeds of motor vehicles, congestion: pedestrians, course of the route*, and *red lights* in an MRA with *conflicts* as the outcome.

Since *ugly–beautiful*, the aesthetical item, was the only predictor that had a relation with both of our main outcomes, it prompted us to explore what it represents. An MRA, with the items from the ACRES as predictors (excluding the two outcome variables), and with *ugly–beautiful* as the outcome, was therefore performed (Model 6).

The variance inflation factor (VIF) was used to check multicollinearity. All models with predictor and outcome variables (and the four background variables) were checked (all values ≤ 3.38, mean: 1.26) and indicated no problem [(22), p. 402]. Possible extreme data cases were identified using Cook's distance. No extreme data cases were found in either of the models (all values ≤ 0.084, mean: 0.004), indicating no problem [(22), p. 383].

The top limit for the inclusion of standardized residuals in the models was set to ± 3 SD. A total of 20 individual cases in twelve models had a standardized residual of more than ± 3. They were, however, included in the simultaneous multiple regression analyses since they were few, and had standardized residuals relatively close to the limit for inclusion, as well as no problems with the Cook's distance. A table with the VIF, the Cook's distance, and the standardized residuals can be found in Table 3.

**Table 3:** The VIF, Cook's distance and standardized residuals.

**Model**	**VIF**		**Cook**		**Standardized residuals** > **(**±**3 SD)**	
	**Mean**	**Maximum**	**Mean**	**Maximum**	***N***	**Maximum**
MRA 1 (Table 6)	1.37	2.13	0.004	0.084	2	−5.17
MRA 2 (Supplementary Table A1)*	1.38	2.24	0.004	0.079	2	−5.21
MRA 3 (Table 7)	1.41	2.17	0.004	0.055	6	−3.44
MRA 4 (Supplementary Table A2)*	1.49	2.17	0.004	0.065	5	−3.55
MRA 5 (Table 8)	1.23	1.54	0.004	0.061	1	3.10
MRA 6 (Table 9)	1.77	3.38	0.004	0.043	–	–
MA 1 [Table 10, predictor variable (X), outcome variable (M)]	1.08	1.15	0.003	0.048	1	3.74
MA 2 [Table 10, predictor variable (X), outcome variable (M)]	1.07	1.15	0.003	0.032	–	–
MA 3 [Table 10, predictor variable (X), outcome variable (M)]	1.08	1.15	0.003	0.029	–	–
MA 4 [Table 10, predictor variable (X), outcome variable (M)]	1.07	1.15	0.003	0.033	–	–
MA 5 [Table 10, predictor variable (X), outcome variable (M)]	1.07	1.15	0.003	0.024	–	–
MA 1–5 [Table 10, predictor variable (M), outcome variable (Y)]	1.07	1.17	0.003	0.053	3	−3.32

N, the number of individuals exceeding ± 3 SD; *Is placed in Appendix.

The values from the MRA are presented as y-intercepts, regression coefficients [unstandardized B, their 95% confidence intervals (CI), and standardized B], as well as adjusted R square (Adj. R^2^) for the overall models. To indicate significance, a statistical level corresponding to at least *p* < 0.05 was used. The values from the MA are presented as the standardized total effect, standardized direct effect, and standardized indirect effect of X on Y.

## 3. Results

### 3.1. Perceptions of the environmental variables in male and female participants

Levels of the predictor variables and the outcome variables are given in Table 4. In the gender comparisons, significant differences were noted in *speeds of motor vehicles* and *congestion: pedestrians*.

**Table 4:** Levels of perceptions and appraisals of the environmental variables in male and female participants (mean, SD, and 95% CI).

	**Outcome variables**		**Predictor variables**										
	**Hinders–stimulates walking**	**Unsafe–safe traffic**	**Exhaust fumes**	**Noise**	**Flow of motor vehicles**	**Speeds of motor vehicles**	**Congestion: pedestrians**	**Conflicts**	**Greenery**	**Ugly–beautiful**	**Course of the route**	**Hilliness**	**Red lights**
Men (*n* = 69)	10.4	11.0	9.30	9.51	9.80	8.90	4.93	6.54	8.42	10.7	5.13	4.09	5.67
	3.04	3.18	3.45	3.34	3.30	2.74	3.57	4.02	3.83	2.87	3.60	3.53	4.06
	(9.70–11.2)	(10.2–11.7)	(8.47–10.1)	(8.71–10.3)	(9.01–10.6)	(8.24–9.56)	(4.07–5.79)	(5.57–7.50)	(7.50–9.34)	(10.0–11.4)	(4.27–5.99)	(3.24–4.93)	(4.69–6.64)
Women (*n* = 225)	10.4	10.8	9.88	9.98	10.3	9.77*	6.21*	6.00	7.78	10.7	4.84	3.77	6.11
	2.95	3.47	3.45	3.26	3.76	3.15	3.90	3.98	4.33	3.21	3.40	3.26	4.05
	(10.1–10.8)	(10.4–11.3)	(9.43–10.3)	(9.55–10.4)	(9.83–10.8)	(9.36–10.2)	(5.70–6.73)	(5.48–6.53)	(7.21–8.35)	(10.3–11.1)	(4.39–5.28)	(3.34–4.20)	(5.58–6.64)

For the questions associated with the variables, see Table 2. *Significant difference between the sexes (*p* < 0.05).

### 3.2. Correlations between the environmental variables

Correlations between the environmental variables are shown in Table 5. The two highest correlations were between *noise* and *flow of motor vehicles* (r = 0.774) followed by between *noise* and *exhaust fumes* (r = 0.737). With respect to the outcome variable *hinders–stimulates walking*, the two highest correlations were with *ugly–beautiful* (r = 0.633) followed by *greenery* (r = 0.429). With respect to the other outcome variable *unsafe–safe traffic*, the two highest correlations were with *conflicts* (r = −0.331) followed by *hinders–stimulates walking* (r = 0.313).

**Table 5:** Correlation matrix for the environmental variables (r).

**Variable name**	**1**	**2**	**3**	**4**	**5**	**6**	**7**	**8**	**9**	**10**	**11**	**12**	**13**
1. Hinders–stimulates walking	–												
2. Exhaust fumes	−0.274^*^	–											
3. Noise	−0.328^*^	0.737^*^	–										
4. Flow of motor vehicles	−0.284^*^	0.728^*^	0.774^*^	–									
5. Speeds of motor vehicles	−0.210^*^	0.462^*^	0.549^*^	0.612^*^	–								
6. Congestion: pedestrians	−0.054	0.214^*^	0.279^*^	0.301^*^	0.243^*^	–							
7. Conflicts	−0.140^*^	0.270^*^	0.374^*^	0.354^*^	0.332^*^	0.637^*^	–						
8. Unsafe–safe traffic	0.313^*^	−0.189^*^	−0.238^*^	−0.183^*^	−0.222^*^	−0.172^*^	−0.331^*^	–					
9. Greenery	0.429^*^	−0.291^*^	−0.265^*^	−0.272^*^	−0.175^*^	−0.150^*^	−0.198^*^	0.212^*^	–				
10. Ugly–beautiful	0.633^*^	−0.192^*^	−0.262^*^	−0.212^*^	−0.164^*^	−0.054	−0.091	0.292^*^	0.491^*^	–			
11. Course of the route	−0.248^*^	0.234^*^	0.247^*^	0.242^*^	0.189^*^	0.184^*^	0.349^*^	−0.286^*^	−0.114	−0.209^*^	–		
12. Hilliness	−0.058	0.139^*^	0.078	0.130^*^	0.051	0.046	0.133^*^	−0.120^*^	0.076	−0.016	0.278^*^	–	
13. Red lights	−0.146^*^	0.410^*^	0.285^*^	0.336^*^	0.248^*^	0.276^*^	0.354^*^	−0.241^*^	−0.244^*^	−0.145^*^	0.344^*^	0.236^*^	–

* Pearson's correlation coefficient (r) is significant at the 0.05 level (2–tailed).

### 3.3. Relations between the predictor variables and the outcome hinders–stimulates walking

The results of Model 1 (in which the item *unsafe–safe traffic* was excluded as a predictor) are shown in Table 6. About 45% of the variance of the outcome variable, *hinders–stimulates walking*, was explained by the predictors in the model (Adj. R^2^ = 0.445). The regression equation was: Y = 4.60 + 0.510 *ugly–beautiful* + 0.139 *greenery* + 0.115 *income –* 0.146 *noise* (all *p*-values ≤ 0.017). Model 2, in which the item *unsafe–safe traffic* was included as a predictor, is presented in Supplementary Table A1.

**Table 6:** Model 1; MRA of predictor variables in the ACRES, with *hinders–stimulates walking* as an outcome, and *unsafe–safe traffic* excluded.

**Outcome variable**	**y–intercept (95% CI)**		***p*-value**
Hinders–stimulates walking	4.60 (2.57–6.63)		< 0.000
**Predictor variables**	**Unstandardized B (95% CI)**	**Standardized B**	***p*** **-value**
Noise	−0.132 (−0.221 to −0.044)	−0.146	0.003
Congestion: pedestrians	0.045 (−0.043–0.134)	0.059	0.316
Conflicts	−0.035 (−0.128–0.058)	−0.047	0.457
Greenery	0.098 (0.025–0.171)	0.139	0.009
Ugly–beautiful	0.483 (0.387–0.580)	0.510	< 0.000
Course of the route	−0.078 (−0.163–0.007)	−0.091	0.073
Hilliness	−0.017 (−0.099–0.066)	−0.019	0.693
Red lights	0.034 (−0.039–0.107)	0.046	0.358
Sex*	−0.229 (−0.875–0.417)	−0.033	0.486
Age*	0.013 (−0.012–0.038)	0.046	0.301
Education*	−0.056 (−0.699–0.588)	−0.008	0.865
Income*	0.398 (0.072–0.723)	0.115	0.017

Adj. R^2^ = 0.445; *Background variable.

### 3.4. Relations between the predictor variables and the outcome unsafe–safe traffic

The results of Model 3 (in which the item *hinders–stimulates walking* was excluded as a predictor) are shown in Table 7. About 20% of the variance of the outcome variable, *unsafe–safe traffic*, was explained by the predictors in the model (Adj. R^2^ = 0.185). The regression equation was: Y = 10.2 + 0.215 *ugly–beautiful* − 0.282 *conflicts* (all *p*-values ≤ 0.001). Model 4, in which the item *hinders–stimulates walking* was included as a predictor, is presented in Supplementary Table A2.

**Table 7:** Model 3; MRA of predictor variables in the ACRES, with *unsafe–safe traffic* as an outcome and *hinders–stimulates walking* excluded.

**Outcome variable**	**y–intercept (95% CI)**		***p*-value**
Unsafe–safe traffic	10.2 (7.25–13.1)		< 0.000
**Predictor variables**	**Unstandardized B (95% CI)**	**Standardized B**	***p*** **-value**
Noise	−0.016 (−0.153–0.122)	−0.015	0.822
Speeds of motor vehicles	−0.063 (−0.205–0.078)	−0.057	0.376
Congestion: pedestrians	0.070 (−0.053–0.194)	0.080	0.261
Conflicts	−0.240 (−0.371 to −0.110)	−0.282	< 0.000
Greenery	0.017 (−0.084–0.118)	0.021	0.738
Ugly–beautiful	0.233 (0.099–0.367)	0.215	0.001
Course of the route	−0.115 (−0.234–0.003)	−0.116	0.057
Hilliness	−0.025 (−0.139–0.090)	−0.024	0.674
Red lights	−0.045 (−0.146–0.056)	−0.054	0.381
Sex*	0.151 (−0.751–1.054)	0.019	0.742
Age*	−0.001 (−0.036–0.033)	−0.004	0.937
Education*	0.567 (−0.327–1.461)	0.069	0.213
Income*	0.360 (−0.092–0.812)	0.090	0.118

Adj. R^2^ = 0.185; *Background variable.

### 3.5. Relations between conflicts as an intermediate outcome in relation to relevant predictors

The result of Model 5, in which *conflicts* was the outcome, is shown in Table 8. About 52% of the variance of the outcome variable is explained by the predictors in the model (Adj. R^2^ = 0.518). The regression equation was: Y = −3.19 + 0.535 *congestion: pedestrians* + 0.165 *course of the route* + 0.126 *sex* + 0.109 *speeds of motor vehicles* + 0.108 red lights + 0.102 *noise* + 0.101 *income* (all *p*-values ≤ 0.043).

**Table 8:** Model 5; MRA with *conflicts* as an intermediate outcome in relation to relevant predictors.

**Outcome variable**	**y–intercept (95% CI)**		***p*-value**
Conflicts	−3.19 (−5.35 to −1.03)		0.004
**Predictor variables**	**Unstandardized B (95% CI)**	**Standardized B**	***p*** **-value**
Noise	0.125 (0.004–0.245)	0.102	0.043
Speeds of motor vehicles	0.142 (0.016–0.268)	0.109	0.028
Congestion: pedestrians	0.554 (0.463–0.644)	0.535	< 0.000
Course of the route	0.192 (0.091–0.292)	0.165	< 0.000
Red lights	0.106 (0.018–0.194)	0.108	0.018
Sex*	1.184 (0.384–1.985)	0.126	0.004
Age*	0.013 (−0.019–0.044)	0.033	0.430
Education*	0.230 (−0.573–1.032)	0.024	0.574
Income*	0.472 (0.071–0.874)	0.101	0.021

Adj. R^2^ = 0.518; *Background variable.

### 3.6. Relations between ugly–beautiful and other items in the ACRES as predictors

The result of Model 6, in which *ugly–beautiful* was the outcome, is shown in Table 9. About 26% of the variance of the outcome variable is explained by the predictors in the model (Adj. R^2^ = 0.259). The regression equation was: Y = 9.67 + 0.354 *greenery* − 0.219 *noise* − 0.147 *course of the route* (all *p*-values ≤ 0.011).

**Table 9:** Model 6; MRA with *ugly–beautiful* as an outcome in relation to other items in the ACRES as predictors.

**Outcome variable**	**y–intercept (95% CI)**		***p*-value**
Ugly–beautiful	9.67 (7.34–12.0)		< 0.000
**Predictor variables**	**Unstandardized B (95% CI)**	**Standardized B**	***p*** **-value**
Exhaust fumes	0.105 (−0.045–0.256)	0.116	0.170
Noise	−0.219 (−0.389– to −0.050)	−0.229	0.011
Flow of motor vehicles	0.026 (−0.130–0.182)	0.031	0.740
Speeds of motor vehicles	−0.043 (−0.174–0.089)	−0.042	0.522
Congestion: pedestrians	0.008 (−0.100–0.117)	0.010	0.881
Conflicts	0.090 (−0.024–0.204)	0.115	0.123
Greenery	0.354 (0.274–0.433)	0.477	< 0.000
Course of the route	−0.147 (−0.249 to −0.044)	−0.161	0.005
Hilliness	−0.024 (−0.125–0.077)	−0.026	0.640
Red lights	0.002 (−0.090–0.094)	0.003	0.965
Sex*	−0.284 (−1.076–0.508)	−0.039	0.481
Age*	−0.004 (−0.034–0.027)	−0.012	0.820
Education*	−0.216 (−1.003–0.571)	−0.028	0.590
Income*	0.005 (−0.392–0.402)	0.001	0.980

Adj. R^2^ = 0.259; *Background variable.

### 3.7. Mediation

Mediation analyses were run with *congestion: pedestrians, course of the route, speeds of motor vehicles, red lights*, and *noise* as predictors and *unsafe–safe traffic* as an outcome, and *conflicts*, as a mediator (Table 10). The indirect effect of X on Y was significant in all models and the direct effect was significant in three models.

**Table 10:** Mediation analyses between predictor variables and *unsafe–safe traffic* as an outcome with conflicts as a mediator.

**Model**	**Predictor (X)**	**Mediator (M)**	**Outcome (Y)**	**Standardized total**		**Standardized direct**		**Standardized indirect**		
				**effect of X on Y**		**effect of X on Y**		**effect of X on Y**		
				**b**	***p*-value**	**b**	***p*-value**	**b**	**95% CI**	**% of total effect**
MA 1	Congestion: pedestrians	Conflicts	Unsafe–safe traffic	−0.179	0.003	0.082	0.264	−0.261	−0.366 to −0.159	146
MA 2	Course of the route	Conflicts	Unsafe–safe traffic	−0.286	< 0.000	−0.188	0.001	−0.099	−0.159 to −0.048	35
MA 3	Speeds of motor vehicles	Conflicts	Unsafe–safe traffic	−0.220	< 0.000	−0.113	0.058	−0.107	−0.170 to −0.056	49
MA 4	Red lights	Conflicts	Unsafe–safe traffic	−0.234	< 0.000	−0.124	0.038	−0.111	−0.173 to −0.057	47
MA 5	Noise	Conflicts	Unsafe–safe traffic	−0.238	< 0.000	−0.125	0.036	−0.113	−0.182 to −0.059	47

Covariables included in the analyses were sex, age, education, and income. There were no significant background variables. To test the indirect effect, 5,000 bootstrap samples generated a confidence interval.

## 4. Discussion

This is probably the first study of the relations between pedestrians' perceptions of route environmental predictors and appraisals of two outcomes of their urban commuting route environments: *hinders–stimulates walking* and *unsafe–safe traffic*.

**Figure 5:**
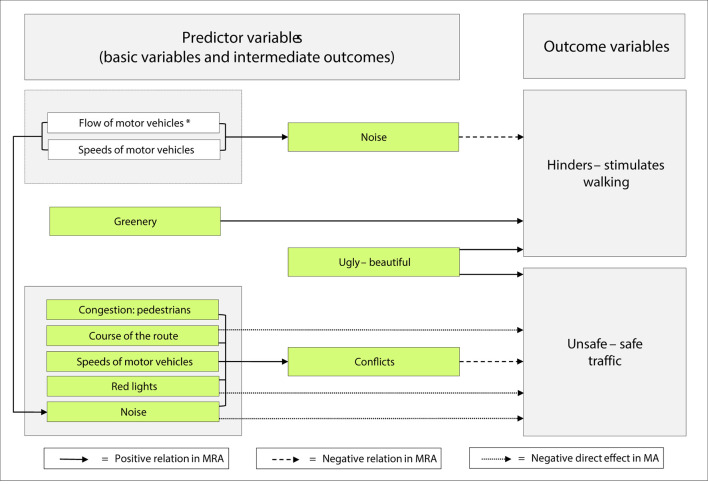
A summary of direct and indirect relations based on commuting pedestrians' perceptions and appraisals of their route environments. In a previous study (23), we have shown that perceptions of both flow and speeds of motor vehicles are related to noise, see * at the box in the upper left corner. MRA = multiple regression analysis; MA = mediation analysis.

Our main results indicate that *ugly–beautiful*, the aesthetical item, and *greenery* appear to strongly stimulate walking, whereas *noise* hinders it. Notably, the standardized Beta coefficients of *ugly–beautiful* and *greenery* add up to 0.65. Furthermore, the aesthetical item is positively related to *unsafe–safe traffic*, whereas *conflicts* has the opposite role. *Conflicts* protrude as the intermediate outcome, representing several basic variables (refer to Figure 5). The mediation analyses demonstrated that some of them also had a direct negative relation with *unsafe–safe traffic*.

### 4.1. The overall models

About 45% of the variance of the outcome variable *hinders–stimulates walking* was explained by the environmental predictors in Model 1 (Table 6) and Model 2 (Supplementary Table A1). About 20% of the variance of the outcome variable *unsafe–safe traffic* was explained by the predictors in Model 3 (Table 7) and Model 4 (Supplementary Table A2). Some of the unexplained variances could be due to missing factors of importance in the ACRES, for example, sidewalks and crosswalks as predictors. Another part of the unexplained variance may be due to the level of reproducibility of the scale (14). In Appendix, comparisons between Model 1 (Table 6) and Model 2 (Supplementary Table A1) as well as between Model 3 (Table 7) and Model 4 (Supplementary Table A2) are commented upon.

In Model 5 (Table 8), where *conflicts* was the outcome, about 52% of the variance of the outcome variable was explained by the predictors. In Model 6 (Table 9), with *ugly–beautiful* as the outcome, about 26% of the variance of the outcome variables was explained by the predictors.

### 4.2. Greenery

Perceptions of *greenery* are positively related to stimulating walking. This is in line with Wahlgren and Schantz (16) who studied commuting cyclists in the same geographical setting. How can this be comprehended? To begin with, humans have a strong preference for natural versus synthetic environments [(24), p. 67; (25, 26)]. Some theories, for example, *Biophilia*, suggest that these preferences arise from early humans evolving in natural environments (27).

Second, exposure to natural settings can reduce stress and anxiety, improve mood, mental health, and psychological functioning (28–30). Walking in an environment with greenery has also been reported to improve well-being during the commute (31).

A third possible explanation is that a green environment may lower the rated perceived exertion during physical activity. This has, at least, been shown when running outdoors in a green and blue environment, compared to on a treadmill in a laboratory setting (32); [cf. (33), pp. 125–126]. The authors hypothesized that outdoor environmental cues “masked” the exertion signals outdoors.

### 4.3. Ugly–beautiful

The aesthetical item is strongly and positively related to *hinders–stimulates walking*. This is in line with Wahlgren and Schantz (16), who studied commuting cyclists in the same geographical setting. In this study, we also noted that the aesthetical item was positively related to *unsafe–safe traffic*.

**Figure 6:**
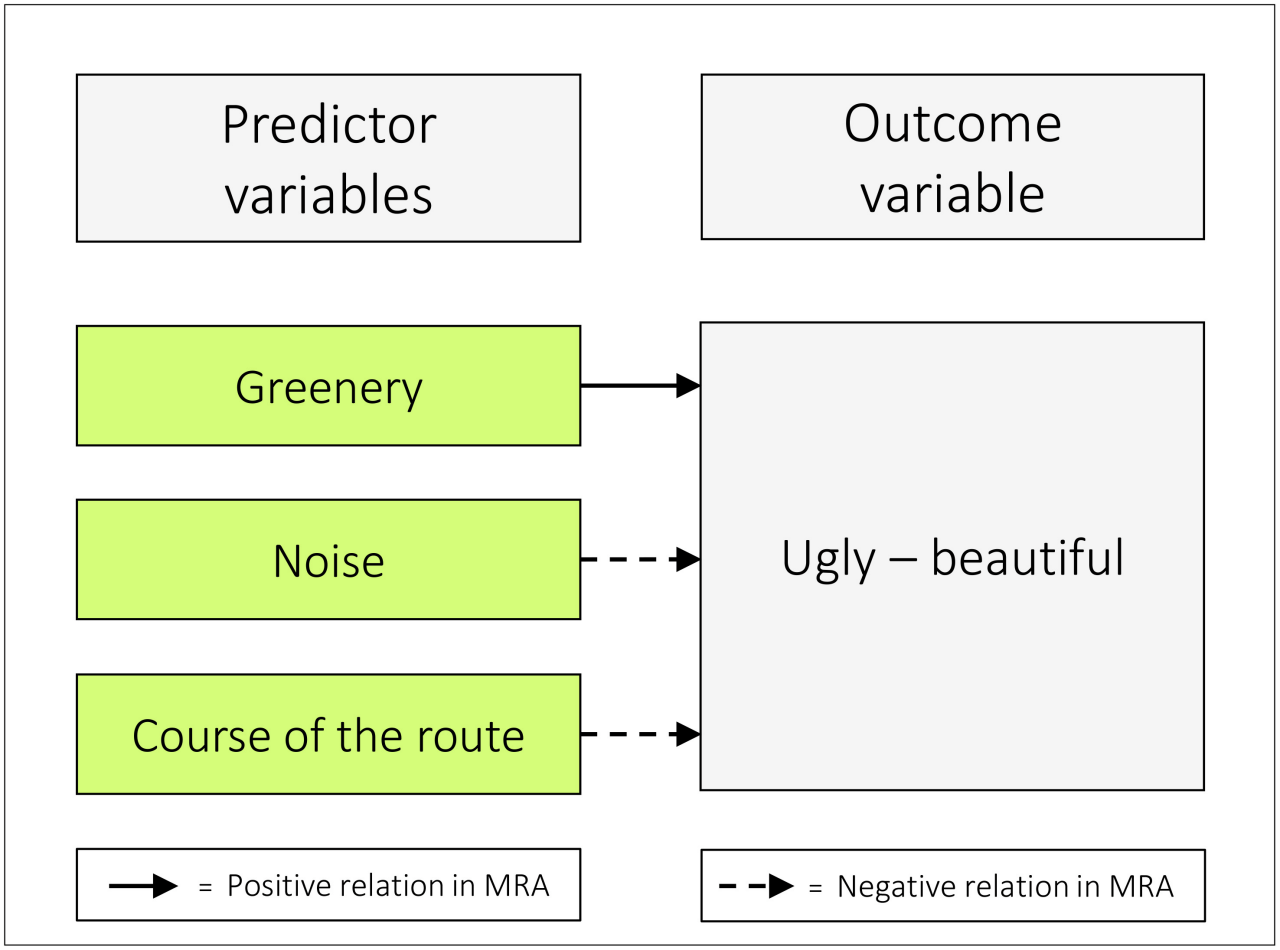
Greenery is positively related to ugly – beautiful, whereas noise and course of the route have the opposite role. MRA = multiple regression analysis.

Given that the variable *ugly–beautiful* influences both of our outcome variables, it is important to thoroughly study the role of this component among those who walk. The fact that the standardized B was 0.51 in relation to *hindering–stimulating walking* illustrates that the influence of the aesthetical item, in itself, is very strong. This raises the intricate question of what the esthetic item really represents. Our separate analysis, among the predictors in ACRES, revealed that *greenery* is positively related to *ugly–beautiful*, whereas *noise* and the *course of the route* have opposite roles (refer to Table 9, Figure 6).

Interestingly, *noise* impacted the aesthetic item negatively. This is in line with research that has shown that auditory stress, such as aircraft noise and human voices, can affect the perceived aesthetic quality of landscapes (34, 35).

In line with our results, the aesthetic preference seems to be amplified by *greenery*, such as trees and flowers (36–40).

Spontaneously, it might seem rather odd that a beautiful environment influences perceptions of safety. However, also other studies indicate that an attractive environment may contribute to a feeling of safety (41, 42). According to Drottenborg [(43), p. 19], it is likely that drivers display lower speed in beautiful compared to in ugly traffic environments. Thus, aesthetically rewarding traffic environments seem to be beneficial for traffic safety, which in such case could be an explanation.

In some studies, *aesthetics* has not been associated with walking for transport (44). In contrast, and in line with our results, *aesthetics* has been reported to be important for transport walking (4). Since the aesthetical item is related to both of our outcome variables, this variable deserves more scientific attention and should be analyzed from different perspectives, in different settings, and among different populations. This is probably a challenge since aesthetic preferences may vary between individuals (45).

### 4.4. Noise

In the present study, perceptions of *noise* were negatively related to *hinders–stimulates walking*. How can this be understood? First of all, *noise* can reach almost everyone in the urban streetscape, regardless of different barriers provided by vehicles, vegetation, or other materials. The World Health Organization (WHO) recommends that noise from road traffic should not exceed 53 dB L_den_, since, above this level, it is associated with negative health effects [(46), p. 30]. In Stockholm County, 30% of the adult population is exposed to noise levels from road traffic exceeding that level at the façade of their dwelling [(47), p. 15].

In line with what our results indicate, long-term transportation noise annoyance can decrease levels of physical activity (48). Proximity to major roads has also been associated with lower levels of physical activity, independently of noise annoyance (48), and it can possibly be due to a barrier effect of traffic, as has been shown in the case of when major roads are located between residential and green areas (49).

The “noise-problem” can only be partially solved with electric cars. There seems to be a threshold at approximately 30 km/h [(50), p. 35, (51), p. 3]. Below this speed an electric car makes less noise than a conventional car (with an internal combustion engine). For speeds above 30 km/h the friction of the tires is the major source of the noise. In urban areas, where the speed often is above 30 km/h [see e.g., (23)] a transformation of the car fleet toward relatively more electric cars, will most likely not have a pivotal influence on the traffic noise.

Furthermore, with respect to road safety, speed management should be a key issue. In accordance with that, a WHO campaign has been launched to limit the speed in urban areas to 30 km/h [(52), p. 60]. This is in line with the present results that *noise* and *speeds of motor vehicles* are negatively related to *unsafe–safe traffic*.

### 4.5. Conflicts—An intermediate outcome

Perceptions of *conflicts*, i.e. the relationships between road users, were negatively related to *unsafe–safe traffic*. As a matter of fact*, conflicts* was the only predictor that was negatively related to *unsafe–safe traffic* in the overall model. Therefore, an MRA with *conflicts* as the outcome and items in the ACRES that were considered to be relevant were chosen as predictors: *noise, speeds of motor vehicles, congestion: pedestrians, course of the route*, and *red lights* (Table 8). The MRA disclosed that all these predictors were positively related to the outcome, that is, all of them are contributing to the perception of *conflicts*. The two predictors that contributed most strongly (*congestion: pedestrians* and *course of the route)* deserve further comments.

*Congestion: Pedestrians* are strongly and positively related to *conflicts*, and this is in line with a study by Van Cauwenberg et al. (4), where places that were crowded were disliked by pedestrians. The inclusion of the item *course of the route* in the ACRES evolved from theories of space syntax (14, 16). *The course of the route* was measured by the question: “To what extent do you feel that your walking trip is made more difficult by the course of the route? For example, a course with many sharp turns, detours, changes in direction, side changeovers, etc.” These characteristics can be recurrently challenging in terms of new encounters with other street users, and novel traffic situations that can lead to *conflicts*. Interestingly, the MA (Table 10) disclosed a negative direct effect between the *course of the route, red lights*, and *noise* as predictors and *unsafe–safe traffic* as an outcome.

Thus, we can conclude that *conflicts* is an intermediate outcome representing several other items in the ACRES.

### 4.6. Strengths and limitations

This study has both strengths and limitations. To begin with, we have studied pedestrian commuters in their route environments. Normally, active commuting is a highly repetitive behavior along a specific route (53). The commuters are, therefore, very familiar with their route environments and can therefore be viewed as “experts.”

Data were collected using ACRES, an instrument that is characterized by considerable criterion-related validity and reasonable test–retest reproducibility (14, 15). The mean of the intraclass correlation coefficient (ICC) regarding the 18 items for cyclists was 0.65 (0.42–0.87). A major advantage of this scale is that it considers the entire route, from home to the workplace. Researchers have emphasized the importance of taking the entire route into consideration when analyzing walking behavior (54, 55). Furthermore, the research was conducted in the inner urban area of Stockholm, Sweden, where pedestrian commuting is a socially accepted behavior. This increases our chances of a diverse study population, although active commuters represent a limited part of the total population.

Another strength is that this study is based on subjective measures of perceptions and appraisals. This is valuable since it is known that a given objective measure can, dependent on individuals' sensitivity, be rated differently. For example, an objective level of noise in dB can induce quite diverse sensations among individuals (56).

With reference to limitations, the advertisement recruitment strategy of the participants could possibly increase the risk of self-selection bias, for example, not everyone is reading newspapers. However, this sampling method has been compared to street recruitment of cyclists regarding ratings of route environments in the same area (15). It was hypothesized that the street recruitment strategy represented the population with greater certainty than the advertisement strategy. Overall, the participants' ratings indicated a reasonably good correspondence between the two groups (15). Furthermore, the characteristics of the two samples, with respect to background factors such as age, gainful employment, and education, showed relatively good agreement [(17), pp. 65–69]. In addition to that, the present participants were characterized by similar levels of gainful employment and income as the pedestrians in the National Travel Survey, who were randomly recruited [(17), pp. 109–110]. Nevertheless, a consequence of possible selection bias is that the results should be interpreted carefully.

## 5. Conclusion

Aesthetics and *greenery* appear to be strong stimulating factors of walking, whereas perceptions of *noise*, as a proxy for traffic, hinder it. Furthermore, aesthetics is positively related to *unsafe–safe traffic*, whereas *conflicts* has the opposite role. *Conflicts* is an intermediate outcome, representing several predictors. *Greenery* is positively related to aesthetics whereas *noise* and the *course of the route* have the opposite role.

Numerous things can be done to improve the quality of the urban environment. A modal shift from individual car use to active commuting is one such example. If policymakers and urban practitioners can focus on the facilitating variables when designing and developing urban route environments, it could possibly be a game changer with respect to public health, attracting new pedestrians, and simultaneously stimulating those who already walk, to keep on walking.

## Data availability statement

The raw data supporting the conclusions of this article will be made available by the authors, without undue reservation.

## Ethics statement

The studies involving human participants were reviewed and approved by the Ethics Committee North of the Karolinska Institute at the Karolinska Hospital, Stockholm, Sweden. The patients/participants provided their written informed consent to participate in this study.

## Author contributions

Conceptualization, supervision, project administration, and resources: PS and LW. Funding acquisition: PS. Visualization, writing—review and editing, writing—original draft preparation, and formal analysis: DA, LW, and PS. Data curation and investigation: DA and LW. Methodology: DA and PS. All authors contributed to the article and approved the submitted version.
