# Supplementary material for: Pedestrians' perceptions of route environments in relation to deterring or facilitating walking
Source: Front Public Health. 2023 Jun 6;10:1012222. doi: 10.3389/fpubh.2022.1012222 (PMC10281529; doi:10.3389/fpubh.2022.1012222)
Supplement: Supplementary file 1 [file Data_Sheet_1.docx]

**Appendix**

In this appendix there are two tables (Tables A1 and A2) describing the relations between predictor variables and the outcome *hinders – stimulates walking* (Table A1, model 2) and *unsafe – safe traffic* (Table A2, model 4). The rationale for performing MRA of these two models is to check for any overlap between what the two outcomes represents, as is presented in the main part of this article. This is enabled by including the outcome *unsafe – safe traffic* as a predictor in model 2, and the outcome *hinders – stimulates walking* as a predictor in model 4.

Comments will be stated regarding differences between model 1 (Table 6 in the main part of this article) and model 2 (Table A1) in Appendix, as well as between model 3 (Table 7 in the main part of this article) and model 4 (Table A2) in Appendix (see below).

*Relations between the predictor variables and the outcome hinders – stimulates walking (model 2)*

The result of the MRA for model 2 (in which the item *unsafe – safe traffic* was included as a predictor) is shown in A1. About 45 % of the variance of the outcome variable, *hinders – stimulates walking*, was explained by the predictors in the model (Adj. R² = 0.450). The regression equation was: Y = 3.82 + 0.490 *ugly – beautiful* + 0.137 *greenery* + 0.106 *income -* 0.142 *noise* (all p-values ≤ 0.027).

Table A1. Model 2; MRA of predictor variables in the ACRES, with *hinders – stimulates walking* as an outcome and *unsafe – safe traffic* included

| Outcome | y-intercept  (95% CI) | | Sign. |
| --- | --- | --- | --- |
| Hinders – stimulates walking | 3.82  (1.64 – 6.00) | | 0.001 |
| Predictor variables | Unstandardized B  (95% CI) | Standardized B | Sign. |
| Noise | -0.129  (-0.217 – -0.041) | -0.142 | 0.004 |
| Congestion: pedestrians | 0.040  (-0.049 – 0.128) | 0.051 | 0.379 |
| Conflicts | -0.015  (-0.110 – 0.079) | -0.021 | 0.749 |
| Unsafe – safe traffic | 0.080  (-0.005 – 0.164) | 0.091 | 0.064 |
| Greenery | 0.097  (0.024 – 0.169) | 0.137 | 0.009 |
| Ugly – beautiful | 0.465  (0.367 – 0.563) | 0.490 | < 0.000 |
| Course of the route | -0.069  (-0.154 – 0.017) | -0.080 | 0.114 |
| Hilliness | -0.015  (-0.097 – 0.067) | -0.016 | 0.725 |
| Red lights | 0.038  (-0.035 – 0.110) | 0.052 | 0.306 |
| Sex* | -0.245  (-0.888 – 0.399) | -0.035 | 0.455 |
| Age* | 0.013  (-0.012 – 0.038) | 0.046 | 0.298 |
| Education* | -0.102  (-0.744 – 0.541) | -0.014 | 0.756 |
| Income* | 0.369  (0.043 – 0.694) | 0.106 | 0.027 |

Notes: Adj. R² = 0.450. * = background variable.

*Comments upon differences between model 1 (Table 6 in the manuscript) and model 2 (Table A1 in Appendix)*

In model 1, with *hinders – stimulates walking* as an outcome, and *unsafe – safe traffic* excluded, *ugly – beautiful* and *greenery* were positively related to *hinders – stimulates walking*. The perception of *noise* was negatively related to the outcome. In model 2, with the same outcome, but including *unsafe – safe traffic* as a predictor, the same predictors as in model 1 were significant and *unsafe – safe traffic* almost reached significance. This indicates that there is a tendency to a certain overlap between the two outcome variables. A change of 20 % or more in standardized B for the environmental predictor variables between models 1 and 2 are commented upon. The only variable reaching this threshold was *conflicts,* which role decreased, indicating that the overlap was related to the relation between *conflicts* and *unsafe – safe traffic*.

*Relations between the predictor variables and the outcome unsafe – safe traffic (model 4)*

The result of the MRA for model 4 (in which the item *hinders – stimulates walking* was included as a predictor) are shown in A2. About 20 % of the variance of the outcome variable, *unsafe – safe traffic*, was explained by the predictors in the model (Adj. R² = 0.185). The regression equation was: Y = 9.46 + 0.147 *ugly – beautiful* - 0.276 *conflicts* (all p-values ≤ 0.043).

Table A2. Model 4; MRA of predictor variables in the ACRES, with *unsafe – safe traffic* as an outcome and *hinders – stimulates walking* included

| Outcome variable | y-intercept (95% CI) | | Sign. |
| --- | --- | --- | --- |
| Unsafe – safe traffic | 9.46  (6.45-12.5) | | < 0.000 |
| Predictor variables | Unstandardized B  (95% CI) | Standardized B | Sign. |
| Hinders – stimulates walking | 0.152  (-0.010 – 0.314) | 0.133 | 0.066 |
| Noise | 0.003  (-0.135 – 0.142) | 0.003 | 0.962 |
| Speeds of motor vehicles | -0.061  (-0.202 – 0.079) | -0.055 | 0.392 |
| Congestion: pedestrians | 0.064  (-0.059 – 0.186) | 0.072 | 0.309 |
| Conflicts | -0.235  (-0.365 – -0.105) | -0.276 | < 0.000 |
| Greenery | 0.002  (-0.100 – 0.104) | 0.003 | 0.965 |
| Ugly – beautiful | 0.160  (0.005 – 0.314) | 0.147 | 0.043 |
| Course of the route | -0.103  (-0.222 – 0.016) | -0.104 | 0.088 |
| Hilliness | -0.022  (-0.136 – 0.092) | -0.021 | 0.705 |
| Red lights | -0.050  (-0.151 – 0.051) | -0.060 | 0.327 |
| Sex* | 0.188  (-0.712 – 1.087) | 0.023 | 0.681 |
| Age* | -0.003  (-0.038 – 0.031) | -0.010 | 0.849 |
| Education* | 0.576  (-0.315 – 1.466) | 0.070 | 0.204 |
| Income* | 0.300  (-0.155 – 0.754) | 0.075 | 0.196 |

Notes: Adj. R² = 0.192. * = background variable.

*Comments upon differences between model 3 (Table 7 in the manuscript) and model 4 (Table A2 in Appendix).*

In model 3, with *unsafe – safe traffic* as an outcome, and *hinders – stimulates walking* excluded, *ugly – beautiful* was positively related, and *conflicts* negatively related to *unsafe – safe traffic*. In model 4, with the same outcome, but including *hinders – stimulates walking* as a predictor, the same predictors as in model 3 were significant and *hinders – stimulates walking* almost reached significance. This indicates that there is a tendency to a certain overlap between the two outcome variables. A change in standardized B for the predictors between model 3 and 4 of 20 % or more is commented upon. Three environmental variables had lowered standardized B of such a magnitude: *noise*, *greenery* and *ugly – beautiful*, which indicates that the overlap was related to the relation between these variables and *hinders – stimulates walking*.
